# Citizen engagement in national health insurance in rural western Kenya

**DOI:** 10.1093/heapol/czae007

**Published:** 2024-02-09

**Authors:** Beryl Maritim, Adam D Koon, Allan Kimaina, Jane Goudge

**Affiliations:** Consortium for Advanced Research Training in Africa (CARTA), P.O. Box 10787, Nairobi 00100, Kenya; Centre for Health Policy, School of Public Health, Faculty of Health Sciences, University of the Witwatersrand, 51 2193, 60 York Rd, Parktown, Johannesburg 2193, South Africa; Health Economics Research Unit, KEMRI-Wellcome Trust Research Programme, 00100, Nairobi 00100, Kenya; Department of International Health, Johns Hopkins Bloomberg School of Public Health, 615 N Wolfe Street, E8143, Baltimore, MD 21205, USA; Health Economics Research Unit, KEMRI-Wellcome Trust Research Programme, 00100, Nairobi 00100, Kenya; Centre for Health Policy, School of Public Health, Faculty of Health Sciences, University of the Witwatersrand, 51 2193, 60 York Rd, Parktown, Johannesburg 2193, South Africa

**Keywords:** Citizen engagement, health insurance, informal workers, Kenya

## Abstract

Effective citizen engagement is crucial for the success of social health insurance, yet little is known about the mechanisms used to involve citizens in low- and middle-income countries. This paper explores citizen engagement efforts by the National Health Insurance Fund (NHIF) and their impact on health insurance coverage within rural informal worker households in western Kenya. Our study employed a mixed methods design, including a cross-sectional household survey (*n* = 1773), in-depth household interviews (*n* = 36), six focus group discussions with community stakeholders and key informant interviews (*n* = 11) with policymakers. The findings reveal that NHIF is widely recognized, but knowledge of its services, feedback mechanisms and accountability systems is limited. NHIF enrolment among respondents is low (11%). The majority (63%) are aware of NHIF, but only 32% know about the benefit package. There was higher awareness of the benefit package (60%) among those with NHIF compared to those without (28%). Satisfaction with the NHIF benefit package was expressed by only 48% of the insured. Nearly all respondents (93%) are unaware of mechanisms to provide feedback or raise complaints with NHIF. Of those who are aware, the majority (57%) mention visiting NHIF offices for assistance. Most respondents (97%) lack awareness of NHIF’s performance reporting mechanisms and express a desire to learn. Negative media reports about NHIF’s performance erode trust, contributing to low enrolment and member attrition. Our study underscores the urgency of prioritizing citizen engagement to address low enrolment and attrition rates. We recommend evaluating current citizen engagement procedures to enhance citizen accountability and incorporate their voices. Equally important is the need to build the capacity of health facility staff handling NHIF clients in providing information and addressing complaints. Transparency and information accessibility, including the sharing of performance reports, will foster trust in the insurer. Lastly, standardizing messaging and translations for diverse audiences, particularly rural informal workers, is crucial.

Key messages

- There is limited engagement of citizens in the National Health Insurance Fund (NHIF) policy formulation and implementation leading to low awareness of benefit NHIF, feedback mechanisms and poor accountability and trust.
- Low engagement contributes to low enrolment and attrition from the national scheme.
- There is need to evaluate current NHIF citizen engagement frameworks to improve citizen accountability and incorporate citizen voice in processes.

## Introduction

Effective citizen engagement initiatives have been proven to be crucial for the success of health insurance systems as they increase awareness of insurance benefits and improve beneficiary satisfaction with the services (Mcintyre, 2010; Ellen *et al*., 2017a; Brito Fernandes *et al*., 2022). As low- and middle-income countries (LMICs) increasingly adopt social health insurance (SHI) programmes to increase population coverage and access to health services, evidence suggests that most schemes suffer growth challenges because of inadequate citizen engagement (Odeyemi, 2014; Kotoh *et al*., 2018). SHI programmes are public health insurance schemes funded through mandatory or voluntary contributions often managed by government (Doethinchem *et al*., 2010). SHI programmes in LMICs are distinct from high-income country (HIC) programmes in that they are predominantly funded by households’ contributions (Chemouni, 2018).

Many terms are used interchangeably to refer to the involvement of the community as users and stakeholders in health systems, e.g. social participation, community engagement, community participation, citizen empowerment, patient participation and stakeholder engagement or participation among others (Smith, 2017; Ogbuabor and Onwujekwe, 2018a; Mbau *et al*., 2020; Arthur *et al*., 2023). Citizen engagement refers to a broad range of actions aimed at ensuring individual or collective citizen participation in design of healthcare delivery programmes through feedback and accountability (IAP2, 2017). In the context of SHI, citizen engagement promotes social accountability as it empowers citizens by enabling them to actively engage in decision-making processes, monitor public services and demand transparency and responsiveness from service providers and the insurance agency. Empowered citizens are better positioned to influence policy, advocate for their rights and demand improved public services (Friis-Hansen and Cold-Ravnkilde, 2013; Danhoundo *et al*., 2018; Feruglio and Nisbett, 2018). Lessons from health insurance programmes also emphasize the central role of citizen engagement in benefit package design to reflect the needs, preferences and values of the population (Center For Global Development, 2017; Nguyen *et al*., 2017; Hanson *et al*., 2019).

Kenya’s social health insurer, the National Health Insurance Fund (NHIF), plays a central role in providing universal coverage in the country. NHIF is a government parastatal established in 1966 and provides coverage for inpatient and outpatient care in accredited public and private health facilities. Currently, it covers ∼25% of the population in Kenya (KNBS and ICF, 2023). While membership into the national universal health coverage (UHC) scheme is mandatory for formal sector employees through income-related payroll deduction, it remains voluntary for informal workers, who can join through the payment of a monthly flat rate of Ksh. 500 per household. The NHIF’s UHC scheme covers a wide range of services in the benefit package (Box 1) (NHIF SUPA Cover Products).

Box 1. NHIF benefit packages

- Outpatient services—consultation, laboratory, investigations, daycare procedures, drugs and dispensation, health education, wellness and counselling, physiotherapy services, immunization, and vaccines as per the KEPI schedule
- Inpatient services
- Maternal care—antenatal and prenatal care and deliveries (normal delivery and caesarian section)
- Reproductive health services: family planning
- Renal dialysis
- Overseas treatment for specialized surgeries not available locally
- Rehabilitation for drugs and substance abuse
- All surgical procedures including transplants
- Emergency road evacuation services
- Radiology imaging services (X-rays, CT scan and MRI)
- Cancer treatment Source: NHIF website

Increasing health insurance coverage through SHI is central to the Kenyan government’s UHC plans. The government recently unveiled a UHC strategy with a focus on primary healthcare and the establishment of a National Social Health Insurance Fund (NSHIF) to replace the NHIF (Government of Kenya, 2023). Indeed, these recent UHC reforms have been aimed at increasing coverage among the informal workers. Less than 8% of NHIF members are informal workers despite being a majority constituting ∼80% of Kenya’s workforce (Shepherd, 2018; Government of Kenya (GoK), 2022). The recent Kenya Demographic Health Survey 2022 indicates that enrolment remains highly inequitable with 17.5% of rural populations having NHIF compared to 35.5% in the urban population (KNBS and ICF, 2023).

While previous studies have explored the level of citizen engagement in NHIF, there has been little focus on rural informal workers despite evidence of the marginalization of this population in access to health services (HEFREP, 2019). Given the national focus on informal workers, this paper therefore seeks to assess citizen engagement by NHIF and the impact on health insurance enrolment among rural informal workers in Kenya.

This study uses the participation, inclusion, transparency and accountability (PITA) framework as a guiding framework for analysing citizen engagement. The framework was synthesized by ) from studies across disciplines related to social accountability, policy and governance where several studies have used one or more of the PITA constructs (Aston *et al*.; UN, 2013; Macaulay *et al*., 2022; Wetterberg *et al*., 2023). By situating our study within the existing literature on citizen engagement, we aim to contribute to the theoretical understanding and practical implementation of citizen-centred approaches in SHI. Table 1 integrates the PITA framework to the SHI role (resource mobilization, pooling and purchasing) and the related actions.

**Table 1.** Integrating PITA framework with SHI roles and related actions

	PITA framework	SHI role and actions
1	Participation: The intervention promotes or formalizes continuous citizen input in the design and implementation of public services, processes or policies	Purchasing: Assessing the health service needs, preference and values of the population and use to specify service entitlement/benefits
2	Inclusion: Includes strategies to promote the opportunities and capacities of marginalized and vulnerable groups such as women and ethnic minorities to engage with the management of public institutions and service providers	Pooling: Inclusion of vulnerable and marginalized groups and ensuring equity in enrolment
3	Transparency: Involves the disclosure and/or dissemination of information (rules, plans, processes, prices and actions) regarding public services or institutions, with the explicit aim of changing the way that citizens and service providers or public officials interact and the power relations between the institution and users	Resource mobilization: Sensitization of citizens to create awareness of the programme
		Pooling: Increasing enrolment to grow the SHI pool for adequate risk and income cross-subsidization
		Purchasing: Inform the population of their entitlement and obligations
		Purchasing: Publicly report on the use of resources and other measures of performance
4	Accountability: Encompasses monitoring and state accountability mechanisms to encourage or actively hold individuals, public service providers and institutions responsible for executing their powers and mandates according to a certain defined standard	Purchasing: Establish effective mechanisms to receive and respond to complaints and feedback from the population
		Purchasing: Ensure equitable distribution of accredited facilities to ensure that population can access their entitlement
		Purchasing: Monitoring providers to ensure provision of services of acceptable quality

## Methods

### Study design

We used an explanatory mixed methods approach combining a quantitative cross-sectional household survey with qualitative interviews and focus group discussions (Creswell, 2014). The survey was embedded in a larger study conducted at the Academic Model Providing Access to Health Care (AMPATH) in western Kenya conducted between August and October 2021.

### Study setting

Data were collected from households in Bunyala subcounty, in Busia County, Kenya. Busia is located on the western border with Uganda where AMPATH implements a UHC pilot that includes household sensitization on NHIF and insurance subsidy for over 2000 vulnerable households (AMPATH, 2022). This study was part of the baseline survey to inform the programme design. AMPATH’s UHC pilot is a partnership between NHIF, Busia County and AMPATH started in 2021 with the goal of testing a scalable model for providing financial risk protection to vulnerable informal worker households in the county (Mercer *et al*., 2018).

The subcounty is made up of 6 administrative locations and 19 sublocations with each location having ∼3000 households. The population is predominantly rural with low socio-economic status as the poverty rate in Busia County is among the highest in the country (66%) (County Government of Busia, 2018a,b). The site is situated on the shores of Lake Victoria, and the main economic activities are fishing and small-scale farming. Approximately 71% of the people working in Busia are employed in subsistence farming, with the remaining 29% working in trading, fishing and other formal and informal jobs. The county’s overall unemployment rate is 66.7% (County Government of Busia, 2022).

### Study participants

Study participants were identified from an AMPATH programme database of all households in Bunyala subcounty provided by local administration. A list of the households was generated for each of the six administrative locations. Stratified random sampling technique was used to select households invited to participate in the study. Locations serve as the second smallest administrative divisions in the country and were utilized as the basis for selecting households for the survey. A sample was chosen from each location, and the sample size was then allocated proportionally to each sublocation within the respective location. This yielded a sample size of 2234 including a 10% adjustment for non-response. The eligibility criteria for the household respondent were household members aged >18 years and who reported having sufficient information about household health utilization.

We identified a subsample of households that participated in the household survey to participate in in-depth household interviews. In each of the six locations, two households were purposively selected from each of the following three groups: currently enrolled in NHIF, previously enrolled and never enrolled to provide multiple perspectives and experiences. Six to eight stakeholders were identified from each of the six administrative locations and comprised local leaders, ward administrators, civil society group members, religious leaders and youth representatives identified for their active role and advocacy in health and community matters. A total of 49 (30 males and 19 females) stakeholders participated in the focus group discussion (FGDs). We also interviewed 11 policymakers and implementers at a national and subnational level purposively selected through snowballing of key informant participants targeting individuals with extensive knowledge and experience in the national health insurance. Policymakers were identified from a diverse institution including academic, development partners (e.g. World Bank) and private sector actors. These were health facility officials, NHIF representatives, researchers, health financing experts and county health officials (Table 2). We observed a point of saturation where new interviews yielded minimal additional information or themes, indicating that a comprehensive understanding of the topic had been reached (Saunders *et al*., 2018).

**Table 2.** KII interview participants

KII participants	No.
National policymakers	5
Insurance experts	1
Development partners	1
County level NHIF representatives	1
Health facility managers	1
Subcounty health management representative	2
Total	11

### Data collection

#### Quantitative

##### Household survey

We used a structured survey questionnaire to train interviewers collecting household data including gender, education, marital status, health insurance status and health status. Interviewers were trained by the research team on key study concepts, ethics and gaining consent as well as the use of handheld devices for data collection. Area chiefs and assistant chiefs were used to identify interviewers based on prior experience in national survey data collection. Community volunteers were engaged to assist data collectors navigate the area and identify the households. Community health volunteers did not participate in the interview process for confidentiality reasons. Each household survey interview was ∼1–1.5 hours. The quantitative data collection tool was designed by the research team and included questions adopted from the national surveys: Kenya Demographic Health Survey and the Kenya Household Health Expenditure and Utilization Survey (Ministry of Health G of K, 2014; National Bureau of Statistics-Kenya and ICF International, 2015). The English questionnaire was translated to Kiswahili and the local language, Luhya, to provide respondents with language options for response.

#### Qualitative

##### In-depth interviews and focus group discussions

Household in-depth interviews (IDIs) took place at the respondent’s houses. FGDs were conducted in local health facilities or community spaces, while key informant interviews (KIIs) were conducted in the interviewees’ preferred location and mostly took place in their respective offices and meeting rooms. Qualitative interviews were conducted by four members of the study team trained in qualitative interviewing. Household indepth interviews and key informant interviews took an average of 30–40 minutes each, while the FGD ran between 2 and 2.5 hours. The study team had debriefing sessions between interviews to report challenges and improve the interviewing process.

Development of the interview guides was informed by the roles of the SHI in health financing as well as its agency role to its citizens (Etienne *et al*., 2010; Ifeagwu *et al*., 2021). This was guided by literature on best practices for developing qualitative interview guides (Stangor, 2011). Using the conceptual framework constructs ensured that we addressed key aspects related to citizen engagement in participation in benefit package design, awareness of entitlements, access to benefit package and awareness of feedback and accountability mechanisms. All data collection tools were pretested a small sample of participants who had similar characteristics to our target population and modifications made to address problems and potential errors.

### Data analysis

#### Analytical framework

For this study, we adopted the PITA framework to assess citizen engagement among rural informal workers in Kenya. The constructs in the framework are widely used to assess citizen engagement in the governance of public institution and delivery of public services (United Nations Development Programme [UNDP], 2011; Waddington *et al*., 2019). We incorporated the constructs as outlined in Table 1. The construct of inclusion was not utilized in this study. This is because the ‘inclusion’ construct within the PITA framework primarily pertains to the involvement of marginalized or disadvantaged groups in decision-making processes, and this was not explored in the scope of our study. Nonetheless, by employing the PITA framework, we can comprehensively assess the level of citizen engagement in SHI, identifying strengths and weaknesses in each of these four crucial dimensions.

### Qualitative data analysis

Audio recordings from the IDIs, FGDs and KIIs were translated (where necessary) and transcribed verbatim by experienced transcribers who were fluent in the languages but not part of the study team. Transcriptions were compared against their corresponding audio files for accuracy. The author read the interview transcripts to familiarize with the data. Codes were developed jointly by the author and two co-authors through a process of identifying key phrases and ideas from a selection of transcripts. The author coded the remaining transcripts using identified codes. The transcripts were coded both deductively and inductively. Initially, we applied an inductive coding approach, allowing us to immerse ourselves in the data and identify emerging themes without imposing preconceived notions. This initial inductive coding phase enabled us to gain a comprehensive understanding of the key themes present in the data and enabled us to identify a suitable framework to structure our analysis. Subsequently, we adopted a deductive coding approach using the PITA framework, which best aligned with the underlying structure and concepts of our data. With this chosen framework as a guide, we conducted a second round of coding, organizing and categorizing the data elements within the established PITA framework. Organization and management of data were done using NVivo 12 software (Gale *et al*., 2013). Lastly, we interpreted findings under each theme and included supportive quotes from interviews.

### Quantitative data analysis

We first conducted descriptive analysis to assess citizen awareness of NHIF, the benefit package, knowledge of feedback and performance accountability mechanisms by household characteristics identified from literature. We then used bivariate logistic regression to examine the influence of household characteristics where the dependent variable was binary if the household respondent (1) was aware of the NHIF cover and (2) knew the benefit package. Odds ratios (ORs) with their corresponding 95% confidence intervals were used to assess the strength of the associations between household factors and the outcome variables (Fenenga *et al*., 2015). Associations between household characteristics and NHIF member satisfaction, awareness of feedback mechanisms and accountability mechanisms were tested using Pearson’s chi square test. Data analysis was done using R and relevant R packages.

### Ethical consideration

Prior to beginning the study, Institutional Research Ethics Committee (IREC) approval was obtained from Moi University/Moi Teaching and Referral Hospital (number: 0003935) and the authors’ institute.

## Results

A total of 1773 households participated in the quantitative household survey with 64% of the respondents being female. On average, each household consisted of five members, and the average age of the participants was 47 years. Table 3 presents the demographic information of the study participants by health insurance enrolment status, i.e. with insurance and without insurance. Among the respondents, 11% reported having NHIF coverage.

**Table 3.** Study participants’ sociodemographic characteristics by insurance status

Have health insurance			
Characteristics	Overall, *N* = 1773	No, *N* = 1568	Yes, *N* = 205
age			
18–24	82.0 (4.6%)	81.0 (5.2%)	1.0 (0.5%)
25–44	790.0 (44.6%)	692.0 (44.1%)	98.0 (47.8%)
45–64	608.0 (34.3%)	524.0 (33.4%)	84.0 (41.0%)
>64	293.0 (16.5%)	271.0 (17.3%)	22.0 (10.7%)
Gender			
Female	1129.0 (63.7%)	1011.0 (64.5%)	118.0 (57.6%)
Male	644.0 (36.3%)	557.0 (35.5%)	87.0 (42.4%)
Marital status			
Divorced/separated	148.0 (8.3%)	137.0 (8.7%)	11.0 (5.4%)
Married/ living together	1106.0 (62.4%)	951.0 (60.7%)	155.0 (75.6%)
Never married/never lived together	116.0 (6.5%)	110.0 (7.0%)	6.0 (2.9%)
Widowed	403.0 (22.7%)	370.0 (23.6%)	33.0 (16.1%)
Ever attended school			
No	356.0 (20.1%)	334.0 (21.3%)	22.0 (10.7%)
Yes	1,417.0 (79.9%)	1234.0 (78.7%)	183.0 (89.3%)
Household size	4.7 (3.6)	4.7 (3.6)	5.1 (3.1)
Occupation			
Formal employment	43.0 (2.4%)	9.0 (0.6%)	34.0 (16.6%)
Homemakers (stay at home)	286.0 (16.1%)	266.0 (17.0%)	20.0 (9.8%)
Others (specify)	11.0 (0.6%)	9.0 (0.6%)	2.0 (1.0%)
Students	8.0 (0.5%)	8.0 (0.5%)	0.0 (0.0%)
Unemployed/seeking work	112.0 (6.3%)	103.0 (6.6%)	9.0 (4.4%)
Working in informal employment	1313.0 (74.1%)	1,173.0 (74.8%)	140.0 (68.3%)
Wealth quintile			
Q1	350.0 (20.0%)	328.0 (21.2%)	22.0 (10.9%)
Q2	350.0 (20.0%)	327.0 (21.1%)	23.0 (11.4%)
Q3	349.0 (20.0%)	317.0 (20.5%)	32.0 (15.9%)
Q4	350.0 (20.0%)	310.0 (20.0%)	40.0 (19.9%)
Q5	350.0 (20.0%)	266.0 (17.2%)	84.0 (41.8%)
Member financial group chama			
No	1101.0 (62.1%)	1006.0 (64.2%)	95.0 (46.3%)
Yes	672.0 (37.9%)	562.0 (35.8%)	110.0 (53.7%)
Admitted last 12 months			
No	1656.0 (93.4%)	1470.0 (93.8%)	186.0 (90.7%)
Yes	117.0 (6.6%)	98.0 (6.2%)	19.0 (9.3%)
Chronic ailment			
No	950.0 (53.6%)	859.0 (54.8%)	91.0 (44.4%)
Yes	823.0 (46.4%)	709.0 (45.2%)	114.0 (55.6%)

### Participation

#### Participation and in benefit package development

There were divergent views on the need for citizens’ involvement and other stakeholders in benefit package design. According to an NHIF representative, citizen needs were adequately represented in the benefit package and the package was informed by epidemiological trends, the socioeconomic status of the population and included chronic ailments associated with catastrophic events:

*We have looked at Kenya and seen the rise of the non-communicable diseases such as cancer and our current package tries to address more of those current challenges. We have gone to a great extent in response to public demand that we address the areas that Kenyans are struggling with* (KII_11).

In response to the view that the NHIF benefit package was based on the prevalence and burden of healthcare costs, a policymaker dismissed these claims saying that the process was political and not informed by data. The benefit package was viewed by policymakers as a political decision that was not data-informed. Some key informants felt that only top-tier level stakeholders were engaged in the development processes leaving out beneficiaries who are users of the package. Policy decision processes within NHIF were termed as ‘opaque’ and lacking objectivity as they seemed to only respond to Ministry of health’s policy direction and not engaging other stakeholders adequately.

*No one knows what’s happening in NHIF- it is a black box. The directions that are taken are very difficult to justify with regards to public involvement in public participation* (KII_09).

There were also concern that NHIF lacked technical capacity required to develop a data-driven package. Gaps in health facility data and records that would be used to identify common conditions, prevalence of conditions and disease trends also made this challenging. Community stakeholders believed that NHIF did not engage or factor in informal workers’ needs, preferences and values when they designed the ‘*Supa Cover*’ package:

*NHIF makes most of the decisions in the NHIF office in Nairobi. There is no way common mwananchi[citizen] is involved. He is just involved in paying*(R7, FGD B).

*I don’t remember any forum where I have been involved as a citizen. But most of these things—I don’t know where they are decided from. They are just pushed. One day you are told this is the cover and there you are. Maybe we are moving from this amount to this amount, and you have no choice. Involvement, especially as far as decision making is concerned, I don’t know where it’s done, but if at all it is done, then it is very minimal.* KII_03 (County health department representative).

### Transparency

#### Informing members of their entitlements and sources of information

Most respondents are aware of NHIF (Table 4). Nearly two-thirds of the participants had heard of NHIF (63%) (Table 4). There were higher odds of awareness of NHIF among those with insurance (OR 2.92) aged 25–40 years (OR 3.33), male (OR 1.26), married (OR 1.42), those who have ever attended school (OR 2.83), being wealthy (Q5 OR 4.43) and those who are members of financial group (OR 1.84); these associations were statistically significant. Awareness was higher among those who had been admitted to hospital in the 12 months and those who had chronic ailment although these were not statistically significant. There were lower odds of being aware of NHIF among those not formally employed.

**Table 4.** Awareness of NHIF supa + cover, benefit package

		Aware of NHIF supa + cover^a^				Aware of benefits^b^			
		Yes % (*n*)	OR	95% CI	*P*-value	Yes % (*n*)	OR	95% CI	*P*-value
Have health insurance	No	60 (942)	1	1		28 (445)	1	1	
	Yes	81 (167)	2.92	(2.05, 4.27)	**<0.001**	60 (124)	3.86	(2.87, 5.23)	**<0.001**
Age	18–24	63 (52)	2.53	(1.54, 4.24)	**<0.001**	23 (19)	1.37	(0.74, 2.44)	0.3
	25–44	69 (549)	3.33	(2.53, 4.41)	**<0.001**	36 (281)	2.50	(1.81, 3.51)	**<0.001**
	45–64	64 (389)	2.60	(1.95, 3.46)	**<0.001**	36 (216)	2.50	(1.79, 3.53)	**<0.001**
	>64	41 (119)	1	1		18(53)	1	1	
**Sex**	Female	61 (684)	1	1	**0.024**	30 (342)	1	1	**0.032**
	Male	66 (425)	1.26	(1.03, 1.55)		35 (227)	1.25	(1.02, 1.54)	
Marital status	Divorced/separated	61 (91)	1	1		33 (49)	1	1	
	Married/living together	69 (768)	1.42	(0.99, 2.02)	0.051	36 (403)	1.16	(0.81, 1.68)	0.4
	Never married/never lived together	56 (65)	0.80	(0.49, 1.31)	0.4	23 (27)	0.61	(0.35, 1.06)	0.081
	Widowed	46 (185)	0.53	(0.36, 0.78)	**0.001**	22 (90)	0.58	(0.38, 0.88)	**0.010**
Ever attended school	No	42 (151)	1	1		19 (66)	1	1	
	Yes	68 (958)	2.83	(2.24, 3.60)	**<0.001**	35 (503)	2.42	(1.82, 3.25)	**<0.001**
Occupation	Formal employment	81 (35)	1	1		63 (27)	1	1	
	Homemaker	57 (162)	0.30	(0.13, 0.64)	**0.003**	23 (67)	0.18	(0.09, 0.35)	**<0.001**
	Others	45 (5)	0.19	(0.04, 0.78)	**0.022**	36 (4)	0.34	(0.08, 1.30)	0.12
	Students	50 (4)	0.23	(0.04, 1.15)	0.068	12 (1)	0.08	(0.00, 0.54)	**0.027**
	Unemployed	53 (59)	0.25	(0.10, 0.57)	**0.002**	22 (25)	0.17	(0.08, 0.36)	**<0.001**
	Informal employment	64 (844)	0.41	(0.18, 0.85)	**0.025**	34 (445)	0.30	(0.16, 0.56)	**<0.001**
Wealth quintile	Q1	45 (157)	1	1		16 (57)	1	1	
	Q2	61 (214)	1.93	(1.43, 2.62)	**<0.001**	26 (90)	1.78	(1.23, 2.59)	**0.002**
	Q3	64 (222)	2.15	(1.59, 2.91)	**<0.001**	33 (115)	2.53	(1.77, 3.64)	**<0.001**
	Q4	67 (235)	2.51	(1.85, 3.42)	**<0.001**	37 (129)	3.00	(2.11, 4.31)	**<0.001**
	Q5	78 (274)	4.43	(3.20, 6.19)	**<0.001**	51 (177)	5.26	(3.72, 7.53)	**<0.001**
Member financial group chama	No	57 (631)	1	**1**		28(309)	1	**1**	
	Yes	71 (478)	1.84	(1.50, 2.26)	**<0.001**	39(260)	1.62	(1.32, 1.98)	**<0.001**
Admitted last 12 months	No	62 (1033)	1	1		32(529)	1	1	
	Yes	65 (76)	1.12	(0.76, 1.67)	0.6	34 (40)	1.11	(0.74, 1.63)	0.6
Chronic ailment	No	62(591)	1	1		31 (291)	1	1	
	Yes	63 (518)	1.03	(0.85, 1.25)	0.8	34 (278)	1.16	(0.95, 1.41)	0.2
	Total	**63 (1109)**				**32 (569)**			

P < .05 in bold

a Have you ever heard of NHIF (National Hospital Insurance Fund)?

b Do you know what services are covered in the NHIF supa + cover?

However, only 32% of the respondents claimed that they were aware of the NHIF benefit package. Among those who were aware of the NHIF benefit package, 61% were covered by NHIF, while 38% did not have an NHIF coverage. These findings highlight the relationship between awareness and insurance coverage, indicating that there is room for improvement in raising awareness even within the NHIF-insured population. Awareness of the benefit package was higher among the insured (OR 3.86), those aged between 25–44 and 45–64 years (OR 2.50), male (OR 1.25), married (OR 1.16), those who had ever attended school (OR 2.42) and those who are members of financial group (OR 1.62). Awareness of the benefit package was higher among those who had been admitted to the hospital within the last 12 months and those who had chronic ailment although these were not statistically significant. There were lower odds of knowing the NHIF benefit package among those not formally employed. Overall, there was high awareness of NHIF but low awareness of the services covered:

*I hear that it pays for your medical bill, but I don’t know exactly what it pays for* (IDI A_Not_active_01).

**Figure 1.**
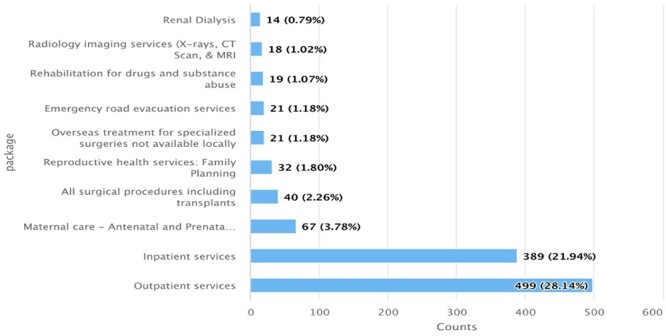
Proportion of respondents who named the services covered by NHIF Supa Cover

NHIF members, who used their card frequently, had better understanding of the benefits. Newly enrolled beneficiaries expected that NHIF covered all healthcare costs indicating that members do not receive comprehensive information on the benefit packages at enrolment. Of the respondents who said that they were aware of the benefit package; we asked them to list the services that were covered by NHIF (Figure 1). The most known services were outpatient and inpatient services with 28% and 21% respondents being aware of these, respectively. The least known services were the renal dialysis and imaging services with only 0.8% and 1.02% being aware. None of the respondents interviewed was able to list all the services covered by NHIF.

*Airlifting is included in the package, but they don’t give the people all details. NHIF needs to inform members that they can be airlifted. It is the smaller details that the people don’t have* (KII_08).

One respondent highlighted the negative consequence of low awareness of NHIF stating:

*Once you are in darkness it is impossible for you to sign up with them* (IDI_A_Active 01).

There was generally low awareness of enrolment procedures, and premium payment channels among the community. There was also confusion regarding beneficiaries to include in the cover with some expecting that extended family members would be covered. Some members reported travelling to the branch offices to make their monthly contributions and were not aware of mobile money payment methods to pay premiums. Low awareness allowed false information regarding NHIF to thrive:

*Sometimes people spread false stories. People can say that they pay one thousand shillings, yet that is not even the amount they pay. One would say, ‘if it is one thousand shillings, I am not joining.’ It would be better if NHIF talked about it themselves then we would know*(IDI_C_Never 01).

According to policymakers, informing members of their entitlements and obligations was the right of contributors. Although NHIF conducted numerous awareness campaigns that created widespread awareness of the NHIF brand, they did not adequately teach people on benefits. Most members only learned of services covered while accessing care:

*Creating awareness is very important, because when I am contributing something, I need to know what the benefits are. We have cases where a client with NHIF goes for a surgery not knowing how much NHIF would cover. During discharge, they are told NHIF will only pay a small part of the total bill. And the client says: ‘I thought NHIF is paying for everything? This is because there is lack of information* (KII_06).

Participants also reported having less access to information compared to urban populations:

*The bad thing is that we live in the outskirts so sometimes the message can be passed and not get to us in the interior* (IDI_B_Active_02).

Respondents reported hearing about NHIF from TV, radio, SMS, print media and sensitization meetings by partners working in the community. The administrative office at the subcounty level reported having organized discussions to engage public members to increase NHIF enrolment in the area. Most community members had neither heard nor participated in such meetings.

#### Transparency in reporting performance

Nearly all participants (97%) were unaware of any accountability reports or forums used by NHIF to report their performance (Table 5). Awareness of accountability mechanisms was higher amongst respondents aged 25–44 years (OR 2.50), those who had ever attended school (OR 2.42) and those with health insurance (OR 3.86). On awareness of these mechanisms, one respondent said: ‘*I don’t know of any. I am in darkness*’ (IDI_A_Active_01). Participants demanded for public accountability, claiming citizens have a right to get information on how NHIF performed in each period on collections, claims, and investments:

*It is a right because they are the contributors of that fund. How do you deny them to know? Is it a secret? Then why do you come for their money?*(KII 08).

**Table 5.** Satisfaction with benefit package, awareness of feedback mechanisms and NHIF financial performance

		Of those insured, % dissatisfied	Feedback mechanisms awareness				NHIF performance reporting mechanisms			
		**% (*n*)**	**No % (*n*)**	**OR**	**95% CI**	***P*-value**	**No % (*n*)**	**OR**	**95% CI**	***P*-value**
Age	18–24	0 (0)	100 (82)	2.53	(1.54, 4.24)	**<0.001**	100 (82)	1.37	(0.74, 2.44)	0.3
	25–44	24 (19)	94 (739)	3.33	(2.53, 4.41)	**<0.001**	97 (765)	2.50	(1.81, 3.51)	**<0.001**
	45–64	35 (25)	92 (560)	2.60	(1.95, 3.46)	**<0.001**	98 (593)	2.50	(1.79, 3.53)	**<0.001**
	>64	21 (4)	95 (278)	1	1		97 (285)	1	1	
Sex	Female	29 (26)	95 (1070)	1	1		98 (1107)	1	1	
	Male	28 (22)	91 (589)	1.26	(1.03, 1.55)	**0.024**	96 (618)	1.25	(1.02, 1.54)	**0.032**
Marital status	Divorced/separated	44 (4)	97 (143)	1	1		99 (146)	1	1	
	Married/living together	29 (36)	92 (1023)	1.42	(0.99, 2.02)	0.051	97 (1069)	1.16	(0.81, 1.68)	0.4
	Never married/never lived together	0 (0)	94 (109)	0.80	(0.49, 1.31)	0.4	97 (112)	0.61	(0.35, 1.06)	0.081
	Widowed	26 (8)	95 (384)	0.53	(0.36, 0.78)	**0.001**	99 (398)	0.58	(0.38, 0.88)	**0.010**
Ever attended school	No	32 (7)	97 (346)	1	1		99 (353)	1	1	
	Yes	28 (41)	93 (1,313)	2.83	(2.24, 3.60)	**<0.001**	97 (1372)	2.42	(1.82, 3.25)	**<0.001**
Occupation	Formal employment	16 (5)	63 (27)	1	1		79 (34)	1	1	
	Homemakers	22 (4)	96 (275)	0.30	(0.13, 0.64)	**0.003**	98 (280)	0.18	(0.09, 0.35)	**<0.001**
	Students	0 (0)	88 (7)	0.23	(0.04, 1.15)	0.068	100 (8)	0.08	(0.00, 0.54)	**0.027**
	Unemployed/seeking work	62 (5)	94 (105)	0.25	(0.10, 0.57)	**0.002**	97 (109)	0.17	(0.08, 0.36)	**<0.001**
	Informal employment	31 (34)	94 (1234)	0.41	(0.18, 0.85)	**0.025**	98 (1283)	0.30	(0.16, 0.56)	**<0.001**
	Others	0 (0)	100 (11)	0.19	(0.04, 0.78)	**0.022**	100 (11)	0.34	(0.08, 1.30)	0.12
Have health insurance	No	-	98 (1537)	**1**	**1**		99 (1560)	**1**	**1**	
	Yes	28 (48)	60 (122)	2.92	(2.05, 4.27)	**<0.001**	80 (165)	3.86	(2.87, 5.23)	**<0.001**
Wealth quintile	Q1	38 (8)	97 (339)	**1**	**1**		99 (347)	**1**	**1**	
	Q2	29 (5)	95 (334)	1.93	(1.43, 2.62)	**<0.001**	98 (343)	1.78	(1.23, 2.59)	**0.002**
	Q3	21 (6)	96 (336)	2.15	(1.59, 2.91)	**<0.001**	98 (341)	2.53	(1.77, 3.64)	**<0.001**
	Q4	44 (14)	94 (328)	2.51	(1.85, 3.42)	**<0.001**	98 (342)	3.00	(2.11, 4.31)	**<0.001**
	Q5	22 (15)	85 (299)	4.43	(3.20, 6.19)	**<0.001**	94 (328)	5.26	(3.72, 7.53)	**<0.001**
Member financial group	No	23 (19)	95 (1048)	**1**	**1**		98 (1078)	**1**	**1**	
	Yes	33 (29)	91 (611)	1.84	(1.50, 2.26)	**<0.001**	96 (647)	1.62	(1.32, 1.98)	**<0.001**
Admitted last 12 months	No	27 (41)	94 (1554)	1	1		97 (1614)	1	1	
	Yes	47 (7)	90 (105)	1.12	(0.76, 1.67)	0.6	95 (111)	1.11	(0.74, 1.63)	0.6
Presence of chronic ailment	No	25 (19)	94 (897)	1	1		97 (923)	1	1	
	Yes	31 (29)	93 (762)	1.03	(0.85, 1.25)	0.8	97 (802)	1.16	(0.95, 1.41)	0.2
		**28 (48)**	**93 (1659)**				**97 (1725)**			

P < .05 in bold

There was significant mistrust in NHIF with respondents expressing concerns about negative media portrayals. Mistrust was fuelled by allegations of fund misappropriation. One respondent referred to a report from an NHIF board member pointing out challenges in the fund:

*Chinua Achebe said that if an alligator comes out of the water and says that the crocodile is sick, will you doubt the story? You cannot doubt the story because the alligator was with the crocodile in water. So, when a board member comes out and says that there are problems in NHIF, there are problems!* (R6_FGD D).

Policymakers felt that NHIF should publish their reports every financial year the way other institutions do consisting of number of enrolees, revenue raised and expenditures. Reporting on the number of enrolees promoted the scheme’s legitimacy and encouraged active members to continue paying and act as an incentive for those not enrolled to join the scheme:

*There is someone who has not registered and thinks that the government wants to eat people’s money and so when they hear how many people are registered and it has helped them, he or she would want to join and be part of them. For some people seeing is believing* (IDI_C_Not_Active_01).

Performance reports on NHIF performance would attract different technical views and opinions to improve the running of NHIF. A policymaker pointed out that the viability of NHIF was in everyone’s interest:

*Everyone wants to make NHIF better because there is a lot at stake. If NHIF collapsed today, the entire health sector will collapse. Everyone’s interest is to see NHIF succeed*(KII_08).

Respondents recommended using physical meetings like annual general meetings to disseminate information on fund utilization. This would also enable instant and direct feedback from citizens instead of sharing through the media. Other beneficiaries recommended using electronic media such as TV programmes for a wider reach and be tailored for different audience.

*Countrywide, the media teams should sit and explain to everyone, ‘this year we have helped this number of Kenyans, and we had this amount of money and we are remaining with this amount in our kitty*(IDI_A_Not_Active_01).

### Accountability

#### Ensuring access to benefit package

Policymakers expressed concern that the NHIF benefit package ‘looked good on paper but when you look closely at implementation, cracks begin to appear’ (KII 04). According to one policymaker, the NHIF benefit package provides comprehensive list of services that were often not accessible to the members:

*A benefit package is not just lists; it’s an entitlement that should result in a particular service being delivered. The issue is not whether the thing was listed. It is whether people receive those services at a particular health facility in the county* (KII_09).

These views of the policymakers were consistent with the complaints from community respondents on availability of quality health services. Public health facilities had staff shortages, frequent stock outs, and patients were often asked to buy supplies like gloves, bleach and cotton swabs despite being covered. A respondent urged members to take responsibility to make inquiries about facilities before selecting their primary care facilities. In some cases, members were informed that some health services were not covered by the NHIF card and were required co-pay:

*I had an experienced whereby I went to the hospital I chose but they still ask for cash for x-ray because they said they don’t use [the NHIF] card in [the] x-ray [department]* (FGD C_R2).

The issue of selecting a primary healthcare facility was raised by many respondents. The lack of portability of services across primary care facilities resulted in respondents incurring out-of-pocket costs:

*I was referred to another hospital which I was told to pay cash because my card could not pay for services at that facility. So, I had to pay more than 10,000 which I didn’t have. This [NHIF] card has not helped me and yet I am a contributor. It is so discouraging*(FGD C_R2).

Others expressed concern that restricting members to one primary care facility was not good because ‘illness doesn’t say I will appear in certain place’ (R8 FGD C).

#### Access to feedback and grievance mechanisms

Nearly all respondents (93%) were not aware of feedback mechanisms (Table 5). There was higher awareness amongst respondents aged 25–44 years (OR3.33), those who had ever attended school (OR 2.83) and those with health insurance (OR 2.92). Of those who were aware, 57% mentioned visiting NHIF offices as the main method of providing feedback, while 40% and 3% use phone calls and email, respectively. Some respondents were aware of some beneficiary engagement platforms. One respondent mentioned a phone-based application that could be used channel grievances; however, most of the respondents were not aware of any feedback mechanisms. Most clients were not aware of NHIF toll-free lines, while those who knew and tried to call the lines claimed that customer care agents rarely pick up. NHIF representatives confirmed that they are experiencing challenges due to a high number of calls and understaffing of the centre resulting in unanswered calls. Those who were privileged to have contact numbers of an NHIF staff could call to ask for assistance:

*The staff give their numbers so that you can contact them using a personal line when you miss them in the office line. So long as you call them during working hours, they will serve you*(FGD D_R2).

Although NHIF had staff based at subcounty facilities, their ability to handle complaints and grievances was limited. Most cases were referred to NHIF branch offices ∼60 km away from the study site forcing beneficiaries to incur transport costs. Complaints followed lengthy and complicated processes, and clients had to make numerous calls and visits. Inability to address complaints and grievances pushed some members to stop paying for NHIF.

*Unless one does follow-ups, they [NHIF] don’t address client issues. Instead, the staff asks them to come on a later date or refer to a senior officer. Sometimes they are forced to go to the regional office and if he’s from the village, that discourages a someone and decides to abandon the issue altogether*(KII 7).

A policymaker added that NHIF should conduct regular customer satisfaction surveys to capture the views of the users of health services in accredited health facilities:

*NHIF does not do customer satisfaction surveys. The health facility staff end up being the ones to give NHIF feedback on their services* (KII_03).

There were also complaints about NHIF members receiving poor treatment at the health facilities. According to policymakers, providers offered poorer services to NHIF members because of delay of reimbursements from the fund and NHIF is unable to hold the facilities accountable for complaints of poor treatment of members because they were guilty of not paying the facilities.

A senior NHIF official admitted the challenges in customer relations stating that there was internal restructuring within NHIF to ensure this was prioritized. Plans included the expansion of the customer call centre and creation of a case management division to track patient complaints in the system.

*There is a very big room for improvement. We would want to engage with our customers and respond to their queries and inquiries. We want to ensure when it comes to the customer experience at the customer call centre, there is no call that’s dropped*(KII_11).

## Discussion

These results reveal high awareness of NHIF but low knowledge on services, feedback and accountability mechanisms. Nearly two-thirds of the respondents were aware of NHIF, but only a third said that they were aware of the benefit package. None of the household survey respondents could identify all the services covered by NHIF in the benefit package. Barely half of the insured were satisfied with the NHIF benefit package but were not aware of mechanisms to reach NHIF for feedback or complaints. Of those who were aware of the feedback mechanisms, the majority (57%) mentioned going to the NHIF office to get assistance. Almost all were not aware of performance reporting mechanisms, but most respondents expressed desire to know about NHIF performance. Negative media reports on NHIF led to mistrust of the fund. Ultimately, poor citizen engagement has resulted in poor enrolment and retention of rural informal workers.

Previous studies conducted in LMICs, such as Nigeria and Mexico, as well as some HICs in Europe, reveal the neglected role of citizen engagement in SHI. These studies have identified the lack of formal frameworks for citizen engagement, which significantly limits citizen’s participation (Chinitz, 2005; Robinson *et al*., 2005; Santana *et al*., 2011; Arredondo *et al*., 2015; Ibe *et al*., 2017; Ogbuabor and Onwujekwe, 2018b; HEFREP, 2019; Marshall et al., 2021). Our findings indicate inadequate participation of rural informal workers in NHIF policy formulation. This is consistent with the previous research, in that the politics of NHIF policymaking prioritizes the preferences of elites while obscuring the voice of a wider array of stakeholders and citizens (HEFREP, 2019; Koon *et al*., 2020). Consequently, in order to develop a benefit package that is responsive to citizens’ needs, it is crucial for the NHIF to establish formal mechanisms that facilitate the inclusion of citizen perspectives in its policy decisions.

Patient and population representation is a means to incorporate citizen voice and participation in SHI programmes (Robinson *et al*., 2005). The United Kingdom and Canada have used citizen juries, panels and dialogues to involve the public and incorporate their preferences in setting health priorities (Robinson *et al*., 2005). While the success of these mechanisms has been attributed to the social and political structures as well as the fiscal capacity of HICs, they can be adopted in LMICs with considerations of potential benefit against the required investment (Ellen *et al*., 2017b). Thailand’s the Universal Health Coverage Scheme (UCS) holds annual public gathering of beneficiaries to inform revisions in the benefit package (Marshall *et al*., 2021). Also, the National Health Security Board that oversees the implementation of the UCS in Thailand is required by law to have 14% citizen representation (including from marginalized groups) in UCS policy development and implementation (Kantamaturapoj *et al*., 2020b). Although studies have reported that these hearings have led to changes in policy, their effectiveness is dependent on the inclusion and representativeness of diverse groups (Kantamaturapoj *et al*., 2020a,). Further research may be needed to test the feasibility of these strategies in the Kenyan context.

There was lack of transparency among respondents about what services NHIF covers in its benefit package and how the package is adjusted. The NHIF package was faulted for not being well defined and changing frequently, leading to respondents having outdated information about NHIF. Our findings show that NHIF members are often unaware of the services covered in accredited health facilities. Previous studies in Kenya reveal varied and inconsistent information regarding what was covered in NHIF national scheme posing a significant barrier to increasing uptake (Barasa *et al*., 2017; HEFREP, 2019). Ultimately, citizens are more likely to hold health insurance agencies accountable if the benefit package is well defined (Klasa et al., 2018).

The strength of this paper is in the mixed methods approach to answering the question on the level of citizen engagement among informal workers. Triangulation of qualitative and quantitative data allowed for a more nuanced insight into the role of citizen engagement in growing coverage of health insurance. Because the goal of this paper was to assess citizen engagement in rural populations, it limits the generalization of the findings in urban areas with better access to information and health services. The study was also conducted in a site with ongoing NHIF sensitization efforts by AMPATH, which may likely influence levels of NHIF awareness among the community.

While we reported that respondents mentioned hearing about NHIF from various sources such as TV, radio, SMS, print media and community sensitization meetings by partners working in the community, we did not assess the breakdown of these information sources. We acknowledge that assessing the specific sources of information would have provided valuable insights into the awareness levels associated with different channels. Additionally, differentiating the impact of ongoing sensitization efforts, such as those conducted by AMPATH during the study period, could have shed light on the awareness levels in similar settings without such interventions. The low awareness observed in our study can be attributed, at least in part, to the sociodemographic challenges faced in rural settings. This finding underscores the importance of developing targeted strategies to overcome these barriers and improve awareness among rural populations.

Lastly, we acknowledge a non-response rate of 20%, which is considered reasonable given the context of the study. The design, ethical considerations and strategies for addressing non-response have been carefully considered to ensure the study’s validity and reliability. We are confident that our research will provide valuable insights within the constraints of this non-response rate.

As Kenya transitions to the NSHIF, these study findings have policy implication in the implementation of ongoing reforms. We therefore make the following suggestions for improving citizen engagement in SHI in Kenya.

- To improve accountability to beneficiaries, the NSHIF should set up clear formal complaint and feedback mechanisms including a well-staffed call centre. Complaints raised about health providers should be adequately addressed as part of NSHIF service monitoring to providers.
- NSHIF should consider setting up mobile community information hubs/camps with NSHIF agents available to provide information and respond to queries. Community hubs should be set up in other central areas to handle feedback and not just at hospitals (to capture complaints unrelated to acute care). While government service centres (Huduma centres) have NSHIF information booths and is an important resource for disseminating information about NSHIF and its benefits to the public, the centres are often in urban centres in big towns making them inaccessible to rural populations.
- NSHIF information desks in health facilities should be empowered to provide information and manage client relations better. Staff manning the NSHIF desks should be adequately trained in customer handling to provide accurate and up-to-date information on NSHIF.
- For campaigns in rural areas, NSHIF should conduct targeted health insurance literacy campaigns to improve citizens’ understanding of the NSHIF’s benefits, coverage and their rights and responsibilities as members. This includes designing tailored educational materials, organizing community workshops and leveraging existing community health structures to disseminate information effectively.
- Lastly, NSHIF should also develop and implement a clear framework on stakeholder engagement.

## Conclusion

There is high awareness of NHIF, but low knowledge on services covered feedback and accountability mechanisms. Limited access to information has led to low awareness and agency among members undermining their ability to advocate for their rightful access to health services. Rural populations were particularly disadvantaged in access to information and their entitlements because of limited access to communication channels used and the pro-urban distribution of health facilities. Lack of transparency on NHIF performance particularly led to mistrust in NHIF and has negative consequences on enrolment and growing NHIF coverage. There is an urgent need to prioritize strategies to strengthen accountability to members by increasing awareness on entitlements, adopting transparent management of SHI and addressing structural barriers to quality healthcare provision.

## Data Availability

Data will be made available upon request.

## Reflexivity statement

The authors include two females and two males with varying experience and qualifications. Three of the authors have extensive experience in qualitative methods and health policy in sub-Saharan Africa, and the third is a biostatistician with a vast quantitative data analysis experience. All authors have extensive experience in conducting household surveys in LMICs.
